# Impact of high dietary cornstarch level on growth, antioxidant response, and immune status in GIFT tilapia *Oreochromis niloticus*

**DOI:** 10.1038/s41598-021-86172-8

**Published:** 2021-03-23

**Authors:** Haojun Han, Zhen Wang, Jiting Wang, Tingting Wang, Yang Li, Dongyan Guan, Huiwen Sun

**Affiliations:** Shandong Provincial Key Laboratory of Animal Biotechnology and Disease Control and Prevention, Laboratory of Aquatic Animal Nutrition and Environmental Health, Shandong Agricultural University,, 61 Daizong Street, Taian City, 271018 Shandong Province China

**Keywords:** Metabolism, Animal physiology, Immunology

## Abstract

This study was conducted to investigate the relationship between different cornstarch levels in tilapia diet and immune function. All test fish were fed with three cornstarch levels: low-cornstarch (0, LS), medium-cornstarch (18%, MS) and high-cornstarch (36%, HS) diets. Three hundred and sixty fish (initial mean body weight 31.73 ± 1.36 g) were randomly allocated into twelve water-circulated tanks, and thirty fish per tank. Compared with the low and medium cornstarch diets, the results of growth showed that the high cornstarch diet significantly decreased the FBW, WGR, and SGR, and increased the FCR of tilapia (*P* < 0.05). The high cornstarch diet significantly decreased the content of crude protein and increased the content of crude lipid in whole body composition (*P* < 0.05). Moreover, the VSI and CF in HS diet were significantly higher than those of LS diet (*P* < 0.05). The results of blood biochemical index exhibited that the HS diet significantly increased the content of blood glucose, and liver/muscle glycogen (*P* < 0.05). The results of antioxidant experiments demonstrated that the content of SOD and T-AOC in MS diet were significantly higher than those of HS diet (*P* < 0.05). Meanwhile, the content of MDA in MS diet was significantly lower than that of HS diet (*P* < 0.05). The results of immune index test showed that the lysozyme activities in the serum, liver, and gill, and the phagocytic activity and index in MS diet were significantly higher than those of HS diet (*P* < 0.05). The challenge assay results revealed that the mortality rate of HS diet was higher than those of LS and MS diets, but the difference was not significant (*P* > 0.05). In conclusion, the overall results suggested that the 36% cornstarch diet reduced not only the growth performance, but also body immunity. Under this experimental condition, GIFT tilapia could tolerate 18% cornstarch, but not 36% cornstarch.

## Introduction

Many studies have shown that the nutritional conditions of fish are closely related to their immune status^1–3^. The nutrients in the feed are not only essential for growth, but also stimulate the immune system of fish^1^. Malnutrition sometimes affects the resistance of fish to diseases, especially in larval and juvenile stages^2^. Previous studies on the effects of feed nutrients on stress and immunity of fish mainly focused on amino acids, fatty acids and micronutrients, and only few studies focused on macronutrients^4,5^. Starch, as a cheap source of feed energy, is often used in fish food to improve the physical properties of feed and replace the energy role of feed protein. Due to abundant supply and low cost, many researchers have studied how to improve the utilisation efficiency of carbohydrate in fish feed. However, it is well known that the ability of digestion, absorption and metabolism of starch in fish, especially in carnivorous fish, is generally limited, and varies with different species^6^. Many results have shown that excessive carbohydrates may cause metabolic disorder and an unhealthy state of fish^7^, and they are speculated to trigger the stress response^8,9^, which may lead to immunosuppression, thus increasing susceptibility to diseases^10^. Some studies have indicated that the herbivorous and omnivorous fish have stronger starch utilisation ability than carnivorous fish^11^. The impacts of high dietary starch level on the immune response of freshwater fish remain unclear, requires further investigation.

Carbohydrate utilisation is affected by many factors, such as dietary habit, growth and development, insulin level, digestive and metabolic enzymes, energy metabolism, carbohydrate type, inclusion dosage, and feeding frequency^6^. The appropriate dietary starch level can save protein and promote the growth and immunity of fish, and the high starch level can cause persistent hyperglycaemia, liver glycogen accumulation, and decreased immune function, thus inhibiting fish growth. However, the mechanism of low carbohydrate utilisation in fish is still unclear. The study investigated the effects of different cornstarch levels on the growth performance, nutritional physiology, and immune function of aquaculture animals, especially freshwater fish. The omnivorous tilapia (*Oreochromis niloticus*) is an important commercial breed in the world, which has the characteristics of strong disease resistance, fast growth and low-cost ingredient feed^12^. Because of its omnivorous and economic importance, GIFT (Genetic Improvement of Farmed Tilapia) tilapia *Oreochromis niloticus* was selected as the experimental fish in the present study. This results will provides basic data and theoretical reference for investigating the correlation between carbohydrate nutrition and immunity of omnivorous fish, and for preparing compound feed.

## Material and methods

### Experimental design and diets

Three diets were formulated to include three cornstarch levels: 0% (low-cornstarch diet, LS), 18% (medium-cornstarch diet, MS), and 36% (high-cornstarch diet, HS) (Table 1). In order to ensure equal energy level, different lipid dosage was added to each group. Before preparing the feed, all the raw materials were crushed and passed through a 60-mesh sieve. All crushed feed materials were mixed evenly according to the feed formula in Table 1, and then corn oil was added. The tiny oil particles were rubbed by hand, and finally distilled water was added to convert the powder feed form into a hard mass. The wet mash was extruded into a 2-mm diameter particle strip by using a small-sized flat die pelletiser, dried naturally, and stored at 4 °C.

**Table 1:** Diet formulation and chemical composition of experimental diets (g/kg dry matter).

Ingredient	Dietary cornstarch levels (%)		
	Low cornstarch	Medium cornstarch	High cornstarch
Fish meal	550.0	450.0	350.0
Soybean meal	100.0	100.0	100.0
Cornstarch	0.0	180.0	360.0
Microcrystalline cellulose	247.0	197.0	147.0
Corn oil	95.0	65	35.0
Vitamin-premix^a^	2.0	2.0	2.0
Mineral-premix^b^	2.0	2.0	2.0
Choline chloride(50%)	4.0	4.0	4.0
Total	1000.0	1000.0	1000.0
**Proximate composition (g/kg dry matter)**			
Crude protein	402.5	337.5	272.5
Total lipid	128.0	92.0	57.0
Crude ash	69.0	57.0	46.0
Gross energy (KJ/g)	15.8	15.3	15.6

^a^Vitamin premix (mg/kg diet): retinol acetate 30 mg; cholecalciferol 5 mg; alpha-tocopherol 60 mg; ascorbic acid 600 mg; vitamin K3 7 mg; thiamin 20 mg; riboflavin 20 mg; pyridoxine HCL 12 mg; vitamin B12 0.05 mg; inositol 100 mg; pantothenic acid 50 mg; niacin acid 35 mg; folic acid 8 mg; biotin 0.06 mg.

^b^Mineral premix (mg or g/kg diet): KI (1%) 60 mg; CoCl_2_·6H2O (1%) 7 mg; CuSO_4_·5H_2_O 20 mg; FeSO_4_·H_2_O 300 mg; ZnSO_4_·H_2_O 200 mg; MnSO_4_·H_2_O 60 mg; Na_2_SeO_3_·5H_2_O (1%) 60 mg; MgSO_4_·7H_2_O 2600 mg.

### Fish and growth experiment

The experimental tilapia were purchased from a local fry farm. Before the formal trial, the fish were domesticated in the controlled water circulation system for two weeks and fed with the basic diet. At the beginning of feeding trial, three hundred and sixty fish (initial mean body weight 31.73 ± 1.36 g) with good health condition were randomly allocated into twelve water-circulated tanks (volume, 400 L), and thirty fish per tank. In this feeding experiment of seventy days, fish was slowly hand-fed to apparent satiation on the basis of visual observation of fish feeding behavior, and the uneaten feed was gathered after every meal by using plastic nets, dried and weighed. The fish were fed three times a day. Water quality parameters included temperature 27.5 ± 3.5 °C, pH 7.3 ± 0.3, dissolved oxygen 5.8 ± 0.4 mg/L, ammonia-N less than 0.05 mg/L, and nitrite-N less than 0.03 mg/L. After the feeding experiment, the fish were fasted for 24 h, the total weight of each aquarium was weighed, the feed intake of each tank was recorded, and the growth performance index of each group of fish was calculated.

### Sample collection and chemical analysis

Before the formal test, twenty fish were selected to analyse the initial whole body composition. At the end of the feeding trial, five fish were randomly selected from each aquarium and frozen (− 20 °C) for whole fish body composition analysis. Another five fish from each aquarium were selected for serum biochemical index analysis. Blood was collected from the caudal vein of fish, and then centrifuged (4000 g at 4 °C for 10 min). The separated serum was frozen at − 80 °C for further analysis. Another five fish each tank were anesthetised and then dissected to obtain the muscle tissue, liver, and gill. Each five fish sample was collected in a bag, indicating the sample number and date. These samples were immediately stored at − 80 °C for further use.

The contents of blood glucose and liver/muscle glycogen were measured through peroxidase and colorimetry respectively. The insulin concentrations were determined following the double antibody sandwich method. All indices were tested using the assay kit of Nanjing Jiancheng Bioengineering Co., Ltd (China) according to the instructions. According to AOAC (2000) method, the content of dry matter, crude protein, crude lipid, and crude ash in whole fish body was analysed.

### Antioxidant-related index assay

Superoxide dismutase (SOD), glutathione peroxidase (GSH-Px), catalase (CAT), total antioxidant capacity (T-AOC) and malondialdehyde (MDA) in the serum of tilapia were analysed using a spectrophotometer. All the indices were tested using the kit of Nanjing Jiancheng Bioengineering Co., Ltd. (China) according to the instructions.

Superoxide dismutase (SOD) activity was measured by its ability to inhibit superoxide anion generated by the xanthine and xanthine oxidase reaction system. One unit of SOD, expressed as unit mL^-1^, was defined as the amount of enzyme that produced a 50% inhibition in colour formation measured at 550 nm. The principle of measuring GSH-Px activity depends on reduction of hydroperoxides by GSH-Px enzyme, thus forming oxidized glutathione, which is recycled to its reduced state by glutathione reductase. One unit of GSH-Px activity was defined as the amount of enzyme that reduces the GSH concentration in the reaction system at 1 µmol/L per min. Total antioxidant capacity (T-AOC) was determined using the ferric reducing antioxidant power assay. The method is based on the reduction of the Fe^3+^ TPTZ complex to the ferrous form at low pH. One unit of T-AOC was defined as a 0.01 increment in the absorbance of the reaction system caused by serum per milliliter reacting at 37 °C for 1 min. The content of Malondialdehyde (MDA) was determined by measuring the absorbance of MDA-thiobarbituric acid (TBA) from the reaction between MDA and TBA at 450 nm.

### Non-specific immune index assay

Lysozyme (LYZ) activities in serum, liver, and gill were measured using the turbimetric method, and the index was tested using the kit of Nanjing Jiancheng Bioengineering Co., Ltd. according to the manufacturer’s instructions.

The phagocytic activity was determined according to Guan et al.^13^. The blood sample was covered on the Histo-paque medium, and centrifuged (3000 g at 4 °C for 10 min). The obtained leukocytes were washed twice in RPMI-1640 medium. The cell suspension (1 mL) was placed on *Saccharomyces cerevisae* suspension and incubated at 37 °C for 1 h. The blend (10 mL) was applied to a clean glass slide and stained with Giemsa solution. Under the biological microscope (Olympus cx22), approximately 200 phagocytes were counted.

### Challenge assay

The challenge assay was carried out according to Guan et al.^13^. In order to evaluate the resistance of experimental fish to *Aeromonas hydrophila*, ten fish from each aquarium were injected with 0.1 mL of a 2.0 × 10^7^ CFU/mL *A. hydrophila*. After being injected, the fish were continued to be fed normally, with carefully monitoring of the status of the fish. Any abnormal behaviour in the fish was recorded, and dead fish were removed at any time. Cumulative mortality occurred in all groups within one week after infection.

### Statistical analysis

SPSS 21.0 statistical software was used. One-way ANOVA and Tukey's test were performed to identify significant differences among groups. The probability of *P* < 0.05 was considered significant.

## Results

### Growth performance

Data related to growth performance and feed utilisation are presented in Table 2. The final body weight, weight gain rate, and specific growth rate of fish fed HS diet were significantly lower than those in LS and MS diets (*P* < 0.05). However, no significant difference was observed between LS and MS groups (*P* > 0.05). Although no significant difference in feed intake was observed among three groups, the feed intake of LS diet was lower than that of MS and LS diets (*P* > 0.05). Moreover, The feed conversion rate in HS diet was significantly higher than that of MS and LS diets (P < 0.05). On protein efficiency ratio parameters, the protein efficiency ratio in LS diet was significantly lower than that of MS and HS diets (*P* < 0.05).

**Table 2:** Growth performance and feed utilization in GIFT tilapia fed test diets*.

Items	Dietary cornstarch levels (%)		
	Low cornstarch	Medium cornstarch	High cornstarch
Initial body weight (g)	31.20 ± 1.51	31.59 ± 1.21	32.40 ± 1.37
Final body weight (g)	71.24 ± 4.36^b^	72.81 ± 3.49^b^	62.61 ± 3.77^a^
Weight gain rate (%)	125.5 ± 11.88^b^	133.37 ± 9.91^b^	93.24 ± 6.05^a^
Specific growth rate (%/day)	1.21 ± 0.13^b^	1.30 ± 0.09^b^	1.05 ± 0.12^a^
Feed intake (g)	66.07 ± 3.12	65.14 ± 3.76	61.63 ± 4.05
Feed conversion rate	1.65 ± 0.15^a^	1.58 ± 0.11^a^	2.04 ± 0.17^b^
Protein efficiency ratio (%)	150.56 ± 6.24^a^	187.49 ± 8.31^b^	179.88 ± 8.55^b^
Survival (%)	100.00	100.00	100.00

*Data represented as mean ± SD of four replicate tanks. Values in the same row with different superscripts are significantly different (*P* < 0.05).Weight gain rate (WGR, %) = (final body weight–initial body weight) × 100/initial body weight; Specific growth rate (SGR %, d^-1^) = 100 × [(Ln (final body weight)—Ln (initial body weight))/duration (60 days)]; Feed conversion ratio (FCR) = feed intake/(final body weight—initial body weight); Protein efficiency ratio (PER,%) = live weight gain (g)/dry protein intake (g).

### Whole fish body composition and somatic parameters

The whole fish body components in all groups at the end of feeding trial are presented in Table 3. Crude protein content in the HS diet was significantly lower than that in the LS and MS diets (*P* < 0.05), but no significant difference was observed between the MS and LS diets (*P* > 0.05). Crude fat content in the HS diet was significantly higher than that in the LS and MS diets (*P* < 0.05), but no significant difference was observed between the MS and LS diets (*P* > 0.05). Moreover, the viscerosomatic index and condition factor in the HS diet were significantly higher than those of LS diet (*P* < 0.05).

**Table 3:** Whole body proximate analysis (g/kg wet basis) and somatic parameters in GIFT tilapia fed test diets*.

Items	Initial^a^	Dietary cornstarch levels (%)		
		Low cornstarch	Medium cornstarch	High cornstarch
Moisture	663.2	625.5 ± 32.9	652.5 ± 53.8	631.8 ± 51.1
Crude protein	145.4	146.6 ± 7.2^b^	139.9 ± 19.6^b^	110.3 ± 26.1^a^
Crude lipid	105.6	108.6 ± 18.5^a^	107.6 ± 18.5^a^	139.2 ± 16.6^b^
Crude ash	72.7	89.3 ± 21.8	90.2 ± 36.1	98.7 ± 22.8
Viscerasomatic index (%)		12.89 ± 0.6^a^	14.55 ± 0.6^b^	16.58 ± 0.7^c^
Condition factor (%)		1.83 ± 0.4^a^	2.43 ± 0.9^b^	2.51 ± 0.6^b^

*Data represented as mean ± SD of four replicate tanks. Values in the same row with different superscripts are significantly different (*P* < 0.05).

^a^Initial values are not included in the statistical analysis.

Viscera index (VSI, %) = 100 × viscera weight/fish weight; Condition factor (CF, %) = 100 × [body weight of fish (g)/length of fish (cm)^3^].

### Blood glucose, and insulin content, and liver and muscle glycogen content

Blood biochemical parameters in three diets are shown in Table 4. Blood glucose content was significantly higher in the HS diet than that in the LS diet (*P* < 0.05), and the blood glucose of HS diet was also higher than that of MS diet, but no significant difference was observed between the HS and MS diets (*P* > 0.05). No significant difference in blood insulin content was observed among all diets (*P* > 0.05). The content of liver and muscle glycogen was significantly higher in the HS diet than that in the LS diet (*P* < 0.05), and the contents of liver and muscle glycogen were also higher in the HS diet than those in the MS diet, but no significant difference was observed between the HS and MS diets (*P* > 0.05).

**Table 4:** Blood biochemical parameters in GIFT tilapia fed test diets*.

Parameters	Dietary cornstarch levels (%)		
	Low cornstarch	Medium cornstarch	High cornstarch
Blood glucose (mmol/L)	5.32 ± 0.71^a^	8.14 ± 1.18^b^	9.02 ± 0.97^b^
Blood Insulin (ng/L)	3.21 ± 0.09	3.19 ± 0.12	3.31 ± 0.15
Hepatic glycogen (mg/g)	6.95 ± 1.03^a^	21.15 ± 1.73^b^	30.03 ± 2.51^b^
Muscle glycogen (mg/g)	1.51 ± 0.16^a^	2.21 ± 0.36^b^	2.42 ± 0.29^b^

*Data represented as mean ± SD of four replicate tanks. Values in the same row with different superscripts are significantly different (*P* < 0.05).

### Antioxidant-related parameters

The antioxidant-related parameters in three diets are shown in Table 5. The SOD and T-AOC activities were significantly lower in the HS group than those in the MS group (*P* < 0.05), but, no significant difference was observed between the LS and MS diets (*P* > 0.05). No significant difference was observed in GSH-Px content among all diets (*P* > 0.05). Meanwhile, the MDA content in the HS diet was significantly higher than that in the MS and LS diets (*P* < 0.05), and no significant difference was found between the LS and MS diets (*P* > 0.05).

**Table 5:** Antioxidant-related parameters of GIFT tilapia fed test diets*.

Parameters	Dietary cornstarch levels (%)		
	Low cornstarch	Medium cornstarch	High cornstarch
Superoxide dismutase (SOD,U/ml)	155.91 ± 11.25^ab^	167.33 ± 11.97^b^	142.11 ± 8.87^a^
Glutathione peroxidase (GSH-Px, U/µl)	1.23 ± 0.05	1.25 ± 0.04	1.19 ± 0.05
Total antioxidant capacity (T-AOC,U/ml)	36.47 ± 2.31^b^	37.62 ± 2.52^b^	29.25 ± 3.03^a^
Malondialdehyde (MDA, nmol/ml)	17.51 ± 2.22 ^a^	15.49 ± 2.43 ^a^	25.93 ± 2.19 ^b^

*Data represented as mean ± SD of four replicate tanks. Values in the same row with different superscripts are significantly different (*P* < 0.05).

### Non-specific immune index

**Figure 1:**
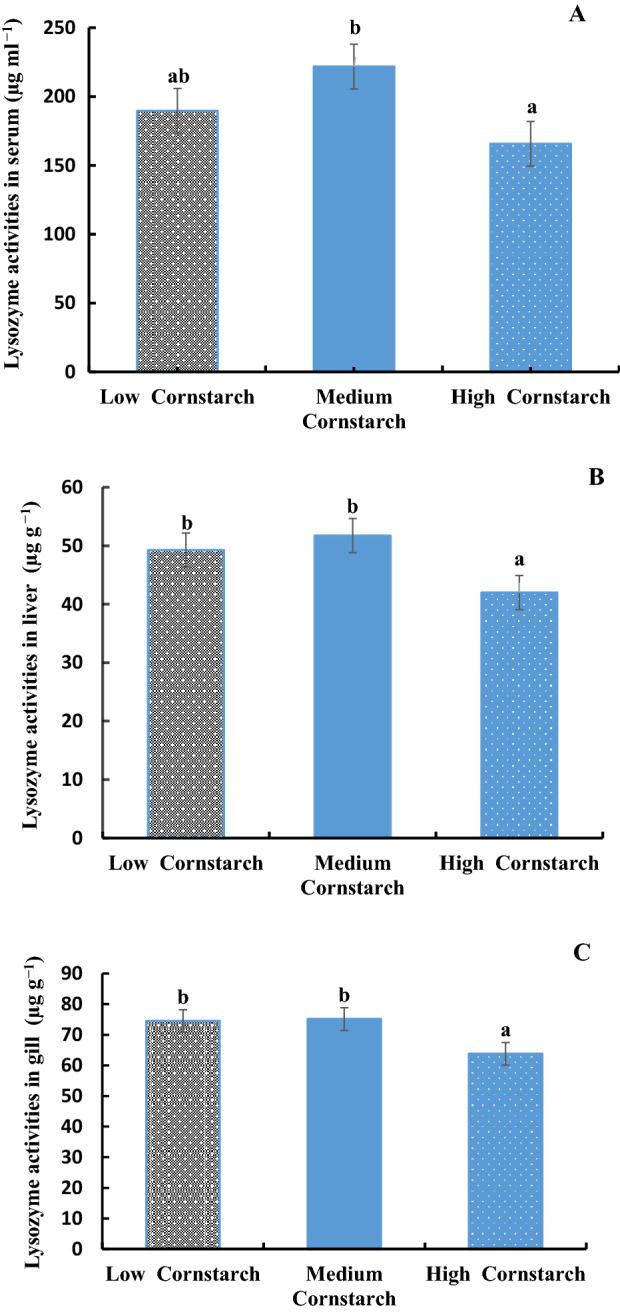
(**A**) Lysozyme activity of blood (μg/ml) in tilapia, Bars with different letters are significantly different (*P* < 0.05). (**B**). Lysozyme activity of liver (μg/g) in tilapia, Bars with different letters are significantly different (*P* < 0.05). (**C**). Lysozyme activity of gill (μg/g) in tilapia, Bars with different letters are significantly different (*P* < 0.05).

**Figure 2:**
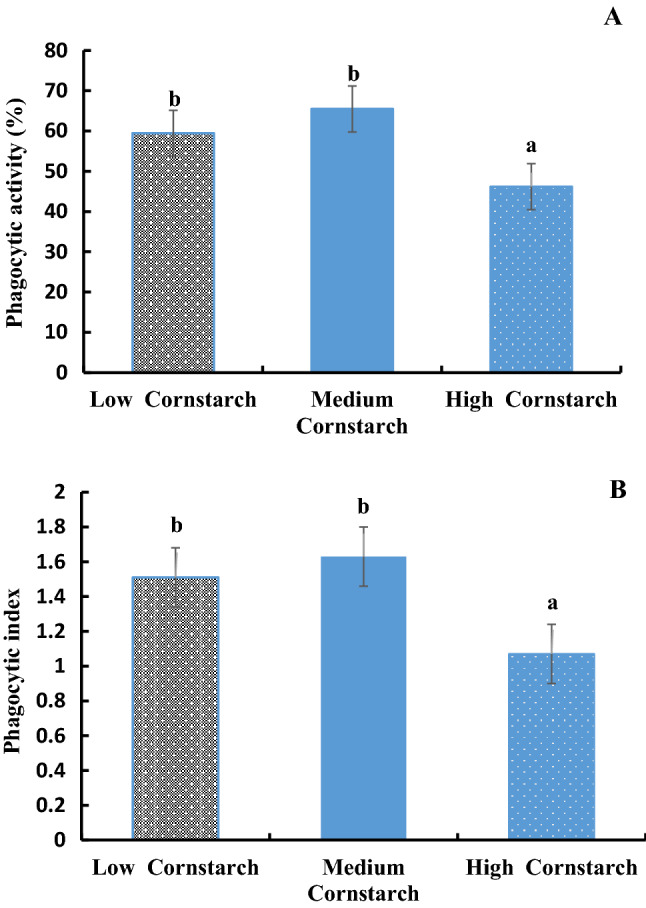
(**A**) Phagocytic activity of leucocytes in tilapia, Bars with different letters are significantly different (*P* < 0.05). (**B**). Phagocytic index of leucocytes in tilapia, Bars with different letters are significantly different (*P* < 0.05). Phagocytosis percentage = no. of ingesting phagocytes/total no. of phagocytes; Phagocytic index = no. of ingested yeast cells/no. of ingesting phagocytes.

The LYZ activities in serum, liver, and gill of tilapia are shown in Fig. 1A–C. These three figures showed that the LYZ activities of serum, liver, and gill in the MS diet were significantly higher than those of the HS diet (*P* < 0.05), but no significant difference was observed between the LS and MS diets (*P* > 0.05). The phagocytic activity and index are presented in Fig. 2. The two figures showed that the phagocytic activity and index in the MS diet were significantly higher than those of the HS diets (*P* < 0.05), and no significant difference was observed between the LS and MS diets (*P* > 0.05).

### Challenge assay

**Figure 3:**
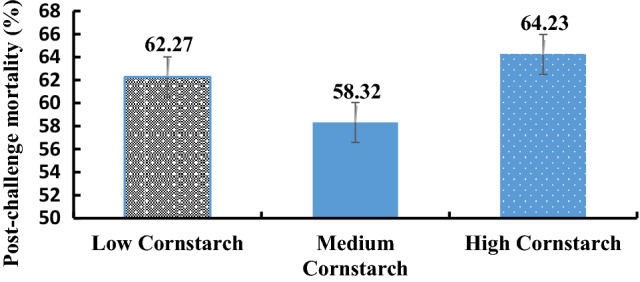
Post-challenge mortality of tilapia after infection with *A. hydrophila* for 7 days.

The post-challenge mortality data for three diets are shown in Fig. 3. The figure showed that the post-challenge mortality in the HS and LS diets was higher than that of the MS diet, however, no significant difference was found (*P* > 0.05). From the data trend, the mortality rate of the HS diet was higher than that of the MS and LS diets (*P* > 0.05).

## Discussion

The current results revealed that high cornstarch level has a significant impact on the growth response of GIFT tilapia, which is similar to the results of other studies^14–17^. Qiang et al.^18^ showed that the WGR and SGR first increased and then decreased with an increase in the carbohydrate level (0, 60, 120, 180, 240 and 300 g/kg) in larval and juvenile tilapias. In addition, Li et al.^17^ found that the specific growth rate decreased linearly with an increase in dietary carbohydrate (52.7, 89.5, 114.9, 143.7, 177.8, 208.2, and 236.5 g/kg). Compared with carnivorous fish, herbivorous and omnivorous fish have a stronger ability to utilise starch. The herbivorous and omnivorous fish can adapt to a certain amount of carbohydrates, showing good growth characteristics, but excessive carbohydrates have a negative impact on fish growth^19^. These results suggest that carbohydrate in feed can be used to meet the energy requirements of fish^20^, which may partly explain the protein-sparing action of carbohydrate in diet. However, the results of Li et al.^21^ in juvenile giant croaker and those of wang et al.^15^ in grouper have revealed that carbohydrate has no protein-sparing action. The reason for the different results of the protein-sparing action of carbohydrate needs to be further explored, and may be related to fish species, diet formulation, water quality, and management condition. In this present study, the high cornstarch diet significantly decreased the content of crude protein and increased the content of crude lipid in whole fish body. The results indicated that with an increase of cornstarch content in feed, the content of crude protein in feed was relatively decreased, leading to an increase of carbohydrate utilisation. This conclusion is consistent with the former findings that the content of crude protein in whole fish body decreases linearly, with an increase of carbohydrate inclusion in the diet, while the content of crude lipid increases quadratically (*P* < 0.05)^17^. The carbohydrate metabolism in fish is mainly carried out in liver, including glycolysis, gluconeogenesis, and glycogen synthesis. Several studies have shown that the viscerosomatic index and condition factor increase with an increase of carbohydrate ratio in the feed of juvenile giant croaker^21^, Wuchang bream^22^, juvenile *Elopichthys bambusa*^23^, and juvenile GIFT tilapia^24^. This showed that fish can convert the excess carbohydrate of feed into body fat, and high carbohydrate intake can enhance fat synthesis and accumulation^25^. The present results showed that the high cornstarch diet significantly improved the viscerosomatic index and condition factor of GIFT tilapia.

In the current study, the datas of blood index showed that high cornstarch level significantly increased the content of blood glucose, and liver/muscle glycogen (*P* < 0.05). The blood glucose is a very crucial index reflecting the glucose metabolism, the physiological state of cells and tissues, and the endocrine function of fish. The ability of carbohydrate digestion and metabolism is poor in fish, especially carnivorous fish^26^. The inhibition of high cornstarch diet on the growth and feed utilization confirmed the glucose intolerance of GIFT tilapia. One of the metabolic pathways of blood glucose in fish is to synthesise glycogen in the liver and muscle. Studies have shown that there was a positive correlation between glycogen deposition and carbohydrate content in feed, and high carbohydrate diet can result in the deposition of more liver glycogen^7^. Although liver glycogen also helps to control the high blood glucose after taking carbohydrate feed, the high content of liver glycogen damages the normal function of the fish liver, resulting in a decline of detoxification ability. Insulin is the only hormone that lowers blood glucose levels. Due to insufficient insulin secretion and regulation, fish are considered to possess a congenital "diabetic constitution". Therefore, excessive carbohydrate may lead to the pathological status of fish, which may inhibit immune function. The present study showed that the blood insulin level was not influenced by different cornstarch levels (*P* > 0.05). This result is consistent with those of previous studies on different types and levels of carbohydrates in *Larimichthys crocea*^27^. It is suggested that the secretion of insulin in fish is not related to the type and level of carbohydrate in feed, and the regulation of blood glucose levels mainly depends on liver glycometabolism-related enzymes and liver glycogen content^28^.

It is well known that the ability of fish to balance blood glucose level is relatively low, so excessive carbohydrates often cause various stress reactions in fish^29^. To prevent oxidative damage, the organism has developed an antioxidant protection system. The antioxidant capacity of organism includes enzymatic and non-enzymatic antioxidant activities, such as T-AOC, GSH-Px and SOD, which are the first defense mechanism against free radicals in organism. Many studies have found that the external defense ability of fish is always affected by feed nutrients^30^; nonetheless, little is known about how the characteristics and content of feed components affect the oxidative capacity of fish. In the current study, feeding tilapia with a high cornstarch diet seems to elicit strong stress symptoms. The results of previous studies on antioxidant stress of juvenile black carp^16^, Wuchang bream^31^, brook trout^32^ , and largemouth bass^33^ fed with high starch diet were similar to those of this study. These studies showed that the higher the activity of antioxidant enzymes, the better the health status of aquatic animals, and the better the metabolic balance^16,22^. At the same time, this study also proved that the excessive dietary cornstarch also reduced the liver antioxidant capacity of tilapia. However, previous studies on carnivorous fish have shown that feed carbohydrates do not cause oxidative stress^34,35^. On the contrary, some studies have pointed out that carbohydrates used as part of the energy have no adverse impact on oxidative stress, or even contribute to oxidative protection^36,37^. Because of different nutritional requirements, formula composition, and carbohydrate addition level, the carbohydrate tolerance of fish is different with different food habits, therefore, many problems still need to be further studied and discussed.

Similar to other vertebrates, aquatic animals have specific and non-specific responses to infectious agents. When fish are attacked by pathogenic microorganisms, the non-specific defense mechanism is more crucial than the specific defense mechanism, because the former is least influenced by environmental temperature, while the latter takes longer to accumulate antibodies and specific cell activation^38^. Therefore, when the external pathogens invade, the non-specific defense system of fish is quickly activated, especially through the production of high reactive oxygen free radical phagocytosis, intracellular killing, and other cellular defense mechanisms, so as to protect fish from pathogens invasion. In recent decades, numerous research results have shown a high correlation between dietary nutrients and body immunity. Better dietary nutrition can improve the immunity of fish, thus helping fish resist external stress and disease^39,40^. Therefore, this study focuses on the humoral and cellular immune indicators that have been proven to be the first line of defense. In the project, we studied the effects of three different cornstarch levels on the immune response and anti-infection ability of tilapia. The results showed that the high cornstarch diet negatively affected the immune response of tilapia. This result is consistent with the findings of Zhang et al.^32^ that excessive dietary carbohydrate intake (> 250 g/kg) may decrease innate immunity status in brook trout. Lysozyme, as an important part of animal immune defense, is often used as an immune index of fish^41,42^. Lysozyme can destroy the polysaccharide wall of bacteria and prevent pathological infection of animals^43^. Zhou et al.^22^ showed that Wuchang bream fed a 310 g/kg carbohydrate diet had significantly increased the lysozyme activity than fish fed a 470 g/kg carbohydrate diet (*P* < 0.05). Likewise, Liu et al.^44^ found that the serum lysozyme activity of fish fed 270 g/kg or 340 g/kg carbohydrate diets was significantly lower than that of fish fed a 140 g/kg carbohydrate diet. In this study, the LYZ activity in serum, liver and gill of fish fed 36% cornstarch was the lowest, which indicated that the high cornstarch diet could reduce the immune ability of tilapia down to a certain level. Phagocytosis is the basic mechanism of the innate immune system of organisms and the first defense line for eukaryotes to resist pathogen invasion^45^. In phylogeny, phagocytosis is the most primitive and basic defense mechanism of all organisms. Phagocytosis is an important index to evaluate the immune status of organisms. It can be used to evaluate how external factors such as diet, temperature and pathogenic bacteria affect the immune function of organism. The most common technique for assessing phagocytosis in vitro is to calculate the phagocytic index. In this study, we determined the immune function of leukocytes isolated from the peripheral blood of GIFT tilapia. The results showed that the phagocytic activity and phagocytic index of tilapia fed high cornstarch diet were significantly lower than that of fish fed medium cornstarch diet. The bacterial challenge test is also often used as an ultimate indicator of fish health, especially to evaluate the health status of fish after feeding certain feed nutrients^4^. As a kind of common gram-negative bacteria, the *Aeromonas hydrophila* can cause hemorrhagic septicemia and ulcerative diseases in animals, and is widely used in the study of nutritional immunity of fish^46^. Previous studies have shown that the mortality is higher in *Labeo rohita* juveniles fed a high carbohydrate diet^3,47^. Wu et al.^16^ also found that feeding juvenile black carp with diets containing carbohydrate (194.3 and 288.4 g/kg) after *A. hydrophila* infection could improve the survival rate. In the present study, the post-challenge mortality in fish fed the high cornstarch diet was higher than fish fed the low cornstarch and medium cornstarch diets, however, the difference was non-significant.

## Conclusion

The current study suggested that the 36% cornstarch diet not only reduced the growth performance of GIFT tilapia, but also increased the content of crude fat in whole fish body, and blood sugar and liver/muscle glycogen level, and lowered the antioxidant and immune indices, thus reducing the body immunity. Under this experimental condition, the GIFT tilapia could tolerate 18% cornstarch, but not 36% cornstarch.
